# Atrial fibrillation as a contributor to the mortality in patients with dementia: A nationwide cohort study

**DOI:** 10.3389/fcvm.2023.1082795

**Published:** 2023-04-03

**Authors:** Yun-Yu Chen, Yenn-Jiang Lin, Yu-Cheng Hsieh, Kuo-Liong Chien, Ching-Heng Lin, Fa-Po Chung, Shih-Ann Chen

**Affiliations:** ^1^ Department of Medical Research, Taichung Veterans General Hospital, Taichung, Taiwan; ^2^ Cardiovascular Center, Taichung Veterans General Hospital, Taichung, Taiwan; ^3^ Cardiovascular Center, Taipei Veterans General Hospital, Taipei, Taiwan; ^4^ Faculty of Medicine, Institute of Clinical Medicine, National Yang Ming Chiao Tung University, Taipei, Taiwan; ^5^ Institute of Epidemiology and Preventive Medicine College of Public Health, National Taiwan University, Taipei, Taiwan.

**Keywords:** atrial fibrillation, cohort, dementia, mortality, risk factor

## Abstract

**Background:**

Knowledge of the risk of death in patients with dementia is essential for planning preventive strategies. This study aimed to evaluate the effect of atrial fibrillation (AF) on death risks and other factors associated with death in patients with dementia and AF.

**Methods:**

We conducted a nationwide cohort study using Taiwan's National Health Insurance Research Database. We identified subjects with dementia diagnosed for the first time and AF diagnosed concomitantly between 2013 and 2014. Subjects under the age of 18 years were excluded. Age, sex, and CHA_2_DS_2_-VASc scores were 1: 4 matched for AF patients (*N* = 1,679) and non-AF controls (*N* = 6,176) using the propensity score technique. The conditional Cox regression model and competing risk analysis were applied. The risk of mortality was tracked till 2019.

**Results:**

AF history was associated with higher risks of all-cause death (hazard ratio [HR]: 1.208; 95% confidence interval [CI]: 1.142–1.277) and cardiovascular death (subdistribution HR: 1.210; 95% CI: 1.077–1.359) in dementia patients than patients without a diagnosis of AF. For patients with both dementia and AF, they had a higher risk of death due to higher age, diabetes mellitus, congestive heart failure, chronic kidney disease, and prior stroke. Anti-arrhythmic drugs and novel oral anticoagulants significantly reduced the risk of death in patients with AF and dementia.

**Conclusion:**

This study found that AF is a risk factor for mortality in patients with dementia and explored several risk factors for AF-related mortality. This study highlights the importance of controlling AF especially in patients with dementia.

## 1. Introduction

Dementia refers to a progressive decline in cognitive ability. In the elderly population (over 60 years), the prevalence of dementia is 5% and doubles every five years after the age of 65 years (1, 2). As the sixth leading cause of death in adults in the United States, dementia represents a major disease burden on life expectancy, with survival estimates ranging from 1 to 15 years (3), and can be influenced by dementia type, clinical risk factors, and cognitive level (4, 5). Developing preventive strategies for patients with dementia requires knowledge of their risk of death.

Atrial fibrillation (AF) is the most common sustained arrhythmia in adults, and its incidence is increasing in the aging population (6). AF is associated with a higher risk of dementia, regardless of stroke history or other shared risk factors (7). Increasing evidence suggests that AF and dementia are frequently associated with the same risk factors for cardiometabolic morbidity (8, 9). In addition to aging, many cardiometabolic factors such as hypertension, diabetes mellitus, heart failure, and smoking status are associated with incident AF and dementia (10). Few studies have examined patients with dementia and a history of AF. This study evaluated all-cause mortality and other factors associated with death in patients with dementia and AF.

## 2. Materials and methods

### 2.1. Study design and study population

**Figure 1:**
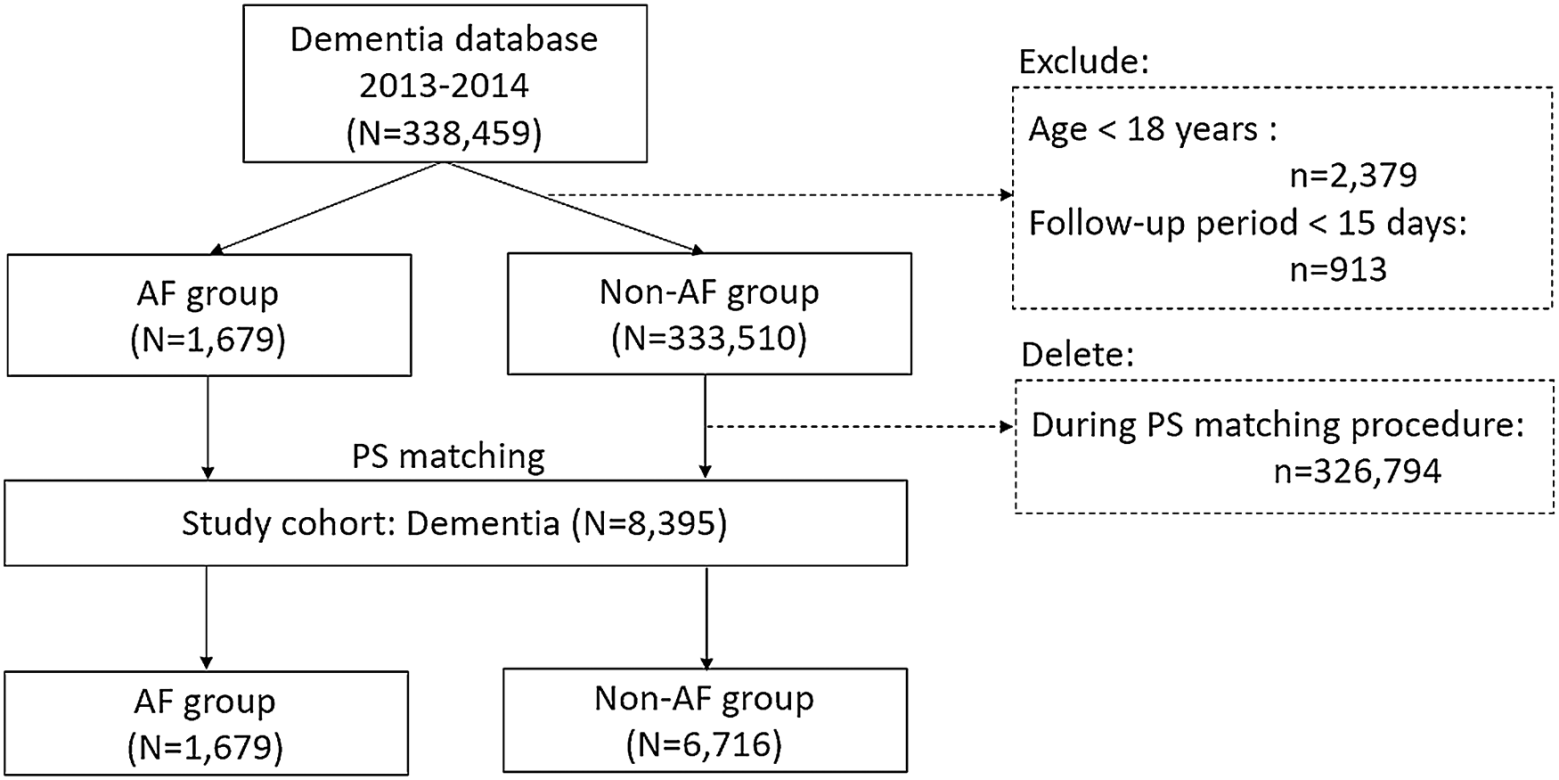
Study flow chart. N or n, number; PS, propensity-score.

We conducted a nationwide cohort study using Taiwan's National Health Insurance Research Database (NHIRD). First, we identified subjects with dementia diagnosed for the first time and AF diagnosed concomitantly between 2013 and 2014 (Figure 1), the index date in this study was set on the date of first diagnosis of dementia. In addition, we reclassified the subjects without a diagnosis of AF at baseline but developed a new AF event during the follow-up period of 2014–2019 into AF group. For those who were reclassified as AF group, the index date was updated to the first date of AF diagnosis thereafter. Subjects under the age of 18 years or with a follow-up period of <15 days were excluded. The propensity score technique was used to match the factors of age, sex, and CHA_2_DS_2_-VASc scores at baseline for AF patients and non-AF controls (1:4 matching) among the 335,189 individuals in the original cohort of dementia. The final database of dementia included 8,395 patients with a diagnosis of dementia, including 1,679 patients in AF group and 333,510 subjects in non-AF controls (Figure 1). This study was approved by the Institutional Review Board (IRB Number: 2021–09-014BC) of Taipei Veterans General Hospital (TVGH) in accordance with the Good Clinical Practice Guidelines.

### 2.2. Ascertainment of baseline data and mortality outcomes from Taiwan's national health insurance research databases

The NHIRDs include records of medical diagnoses during outpatient visits and hospitalizations, medication prescriptions, and medical interventions for >99% of the 23 million Taiwanese population since 1995 (11, 12). The International Classification of Diseases, Ninth Revision/Tenth Revision, Clinical Modification (ICD-9-CM [before 2016]/ICD-10-CM [after 2016]) coding system was used to diagnose the disease (13). To improve the accuracy of coding, the diagnoses were confirmed only if the patient had at least one record of hospitalization or at least three consecutive outpatient visits. Dementia was classified into three sub-types: (1) Alzheimer's disease and senile dementia (ICD-9-CM: 290.0–290.2, 331.0; ICD-10-CM: F00, F03, and G30), (2) vascular dementia (ICD-9-CM: 290.4; ICD-10-CM: F01), and (3) other dementia (ICD-9-CM: 294.1–294.2; ICD-10-CM: F02) (14). The diagnosis of AF was defined as an ICD-9-CM code of 427.31 or ICD-10-CM code of I48 (15). The duration of AF was defined as the number of years between the first AF diagnosis and the date of the death event, if one occurs, or until the end of follow-up if no death event occurs.

Other important clinical variables were identified, including age (years), sex, congestive heart failure (ICD-9-CM: 428, ICD-10-CM: I50), hypertension (ICD-9-CM: 401–405, ICD-10-CM: I10-I15), diabetes mellitus (ICD-9-CM: 250, ICD-10-CM: E08), prior stroke (ICD-9-CM: 430–438, ICD-10-CM: I60-I68), chronic kidney disease (ICD-9-CM: 584–585, ICD-10-CM: N18), chronic obstructive pulmonary disease (ICD-9-CM: 490–496, ICD-10-CM: J44), hyperlipidemia (ICD-9-CM: 272, ICD-10-CM: E78), and thyroid diseases (ICD-9-CM: 242, ICD-10-CM: E05). Between 2013 and 2014, the confirmation for baseline characteristic was conducted on congestive heart failure, hypertension, diabetes, stroke, chronic kidney disease, chronic obstructive pulmonary disease, hyperlipidemia, and thyroid disease, during the same period as the index date. The time window to define the medication uses was tracked from the enrollment to the end of follow-up. The CHA_2_DS_2_-VASc score was calculated based on the presence of heart failure (1 point), hypertension (1 point), age (≥75 years: 2 points), age (65 to 74 years: 1 point), diabetes mellitus (1 point), previous stroke or transient ischemic attack (2 points), female sex (1 point), and vascular disease (1 point) (16). The status of receiving AF ablation was based on (1) an AF diagnosis, (2) the procedural codes of AF catheter ablation (33091 B, 33139 B, 33140 B), and (3) procedural codes for trans-septal puncture (17). Medications were identified using the codes based on the Anatomical Therapeutic Chemical (ATC) classification system. The death record was based on the Death Registry and was followed until the end of 2019.

### 2.3. Statistical analyses

Normally distributed continuous variables are presented as mean ± standard deviation and compared using Student's *t*-test. Continuous variables with a non-normal distribution are presented as median and interquartile range (IQR). Categorical values are presented as absolute numbers (*N*) with percentages (%), and chi-square tests were used for statistical comparisons. Incidence rates of events were calculated as the number of cases per 1,000 person-years (PYs), along with 95% confidence intervals (CIs). The propensity score was applied to match patients with AF and non-AF controls in the ratio of 1 to 4 to minimize the impact on dementia risk due to the imbalanced distribution of age, sex, and CHA_2_DS_2_-VASc score at baseline between patients with AF and non-AF controls. To verify the standardized difference between AF patients and non-AF controls in terms of age, CHA_2_DS_2_-VASc score, and propensity-score, a balance assessment was conducted, Kernel density plot of the propensity score after matching was applied. Kaplan-Meier survival curves were plotted to analyze time-to-event data, and the log-rank test was used to determine statistical significance. Univariable effect and multivariable effect based on conditional Cox proportional hazards regression analysis were used to compare the hazard ratios (HRs) with 95% CIs for mortality outcomes.

Competing risk models (Fine and Gray's method) based on Cox proportional hazard models were used to analyze the subdistribution hazard ratios (SHR for survivors [non-deaths] vs. cardiovascular deaths [CV deaths] vs. non-CV deaths). When competing events such as CV deaths vs. non-CV deaths occur, the competing event may interfere the observation of the event of interest or modify the probability of the event. The competing risk models allow for the separation of events into various causes in order to provide real probabilities of the deaths (18, 19). In this study, cumulative incidence function *via* Fine-Gray subdistribution hazard model was used to estimate the probability of incident CV death. This study also evaluated the regression coefficients (usually represented as beta) to estimate the impact of AF as time-varying covariate (20, 21). To assess the risk factors of mortality, we selected a factor with a *P*-value of ≤0.05 in the univariate analysis and included it in a multivariable analysis. Statistical analyses were performed using SAS version 9.4.

## 3. Results

### 3.1. Characteristics of patient with dementia

A total of 8,395 patients with dementia were studied, including 6,716 non-AF controls and 1,679 patients with AF patients (mean age: 82.5 ± 7.36 years, 47.6% men) (Figure 1). The mean AF duration (years) in dementia patients with AF was 3.74 ± 4.49 years. Table 1 shows the distributions of age and CHA_2_DS_2_-VASc at baseline, and the prevalence of men were similar between non-AF controls and AF patients (refer the baseline characteristics of patients with dementia prior to PS matching in Supplementary Table S1). The Kernel density plot of the propensity score after matching revealed that the distribution of the propensity score between AF patients and non-AF controls was similar (Supplementary Figure S1A). Balance assessment (Supplementary Figure S1B) shows that standardized differences of age, sex, CHA_2_DS_2_-VASc score, and propensity score between AF patients and non-AF controls were 0.030, 0.029, 0.039, and 0.000 in the PS matched dataset. The subtypes of dementia and mortality outcomes between non-AF controls and AF patients are summarized in Table 2. Based on dementia patients with and without AF in the PS-matched, a history of AF had a higher prevalence rate of vascular dementia than non-AF controls (28.7% vs. 17.0% in AF patients vs. non-AF controls, respectively; *P* < 0.001).

**Table 1:** Baseline characteristics of patients with dementia (after propensity-matching).

Baseline characteristics	Patients with dementia (*N* = 8,395)	Non-AF history (*N* = 6,716)	With AF history (*N* = 1,679)	*P*-value
Age at baseline (years)	82.5 ± 7.36	82.5 ± 7.39	82.3 ± 7.22	0.27
CHA_2_DS_2_-VASc	1.71 ± 1.25	1.73 ± 1.25	1.78 ± 1.24	0.15
Sex: male	3,996 (47.6%)	3,216 (47.9%)	780 (46.5%)	0.29
Propensity score	0.993 ± 0.004	0.993 ± 0.004	0.993 ± 0.004	>0.99
Duration of AF (years)	Na	Na	3.74 ± 4.49	Na
Hypertension	2,749 (32.7%)	2,286 (34.0%)	463 (27.6%)	<0.001
Heart failure	298 (3.55%)	117 (1.74%)	181 (10.8%)	<0.001
Diabetes mellitus	1,025 (12.2%)	880 (13.1%)	145 (8.64%)	<0.001
Hyperlipidemia	411 (4.90%)	338 (5.03%)	73 (4.35%)	0.25
Chronic kidney disease	214 (2.55%)	149 (2.22)	65 (3.87%)	<0.001
Stroke	2,379 (28.3%)	1,872 (27.9%)	507 (30.2%)	0.06
Coronary artery disease	31 (0.37%)	20 (0.30%)	11 (0.66%)	0.031
Liver cirrhosis	31 (0.37%)	28 (0.42%)	3 (0.18%)	0.15
Chronic obstructive pulmonary disease	417 (4.97%)	305 (4.54%)	112 (6.67%)	<0.001
Thyroid disease	10 (0.12%)	5 (0.07%)	5 (0.30%)	0.018
Sleep apnea	454 (5.41%)	362 (5.39%)	92 (5.48%)	0.89
Receiving AF ablation	158 (1.88%)	0 (0.00%)	158 (9.41%)	<0.001
Medication uses				
Anti-arrhythmic drugs	743 (8.85%)	325 (4.84%)	418 (24.9%)	<0.001
Beta-blocks	2,610 (31.1)	1,952 (29.1%)	658 (39.2%)	<0.001
Novel oral anticoagulants	1,024 (12.2%)	362 (5.39%)	662 (39.4%)	<0.001
Warfarin	625 (7.44%)	255 (3.35%)	400 (23.8%)	<0.001

AF, atrial fibrillation; N, number; Na, not available.

**Table 2:** Dementia types and mortality outcomes in the dementia cohort dividing by a history of atrial fibrillation or not.

Baseline characteristics	Non-AF history (*N* = 6,716)	With AF history (*N* = 1,679)	*P*-value
Dementia types (*N*, %)			<0.001^a^
Alzheimer disease + senile dementia	5,768 (85.9%)	1,312 (78.1%)	<0.001^a^
Vascular dementia	1,145 (17.0%)	482 (28.7%)	<0.001^a^
Other dementia	930 (13.8%)	261 (15.5%)	0.08^a^
Mortality outcomes			
All-cause deaths (*N*, %)	2,549 (38.0%)	641 (38.2%)	0.87^a^
Incidence rate per 1,000 person-years (95% CI)	78.2 (72.0–84.4)	85.2 (81.9–88.5)	
Model 0: crude effect (HR, 95% CI)	1 (reference)	1.162 (1.139–1.187)	<0.001^b^
Model 1: adjusted effect* (HR, 95% CI)	1 (reference)	1.208 (1.142–1.277)	<0.001^b^
Age < 65 years* (HR, 95% CI)	1 (reference)	5.549 (1.148–26.81)	0.033^b^
Age ≥ 65 years* (HR, 95% CI)	1 (reference)	1.194 (1.128–1.263)	<0.001^b^
	*P* for interaction between age and AF:		0.003^c^
Cardiovascular deaths (*N*, %)	721 (10.7%)	255 (15.2%)	<0.001^a^
Incidence rate per 1,000 person-years (95% CI)	24.1 (22.3–25.8)	31.6 (27.7–35.6)	
Model 0: crude effect^Ɨ^ (SHR, 95% CI)	1 (reference)	1.210 (1.077–1.359)	<0.001^b^
Model 1: adjusted effect*^Ɨ^ (SHR, 95% CI)	1 (reference)	1.185 (1.033–1.358)	<0.001^b^
Age < 65 years* (SHR, 95% CI)	1 (reference)	1.632 (1.007–55.29)	<0.001^b^
Age ≥ 65 years* (SHR, 95% CI)	1 (reference)	1.177 (1.026–1.349)	0.020^b^
	*P* for interaction between age and AF:		0.038^c^

AF, atrial fibrillation; CI, confidence interval; HR, hazards ratio; *N*, number; SHR, sub-distributional hazard ratio.

Model 0, crude effect.

Methods of statistical tests: ^a^Chi-square test, ^b^Cox proportional hazard regression, ^c^interaction analyses.

*Model 1 was adjusted for age, sex, CHA_2_DS_2_-VASc, chronic kidney disease, chronic obstructive pulmonary disease, beta-blockers, antiarrhythmic drugs, novel oral anticoagulants, and warfarin.

^{\hyphen\hskip-3pt{\rm I}}For estimating the SHR, competing risk of cardiovascular death was evaluated using Fine-Gray subdistribution hazard model: Non-death (no death event: 0) vs. cardiovascular deaths (main event: 1) vs. non-cardiovascular deaths (other death event: 2).

### 3.2. Incidence rates and risks of mortality events in patients with dementia

During a median follow-up of 4.68 (IQR: 3.05–6.36) years, the cumulative rates of all-cause deaths were 38.2% vs. 38.0% for AF patients vs. non-AF controls, respectively (*P* = 0.87); the cumulative rates of CV deaths were 15.2% vs. 10.7% for AF patients vs. non-AF controls, respectively (*P* < 0.001) (Table 2). The incidence rates of deaths were higher in AF patients (all-cause deaths: 85.2 per 1,000 PYs, 95% CI: 81.9–88.5 per 1,000 PYs; CV deaths: 31.6 per 1,000 PYs, 95% CI: 27.7–35.6 per 1,000 PYs; respectively) than in non-AF controls (all-cause deaths: 78.2 per 1,000 PYs, 95% CI: 72.0–84.4 per 1,000 PYs; CV deaths: 24.1 per 1,000 PYs, 95% CI: 22.3–25.8 per 1,000 PYs).

**Figure 2:**
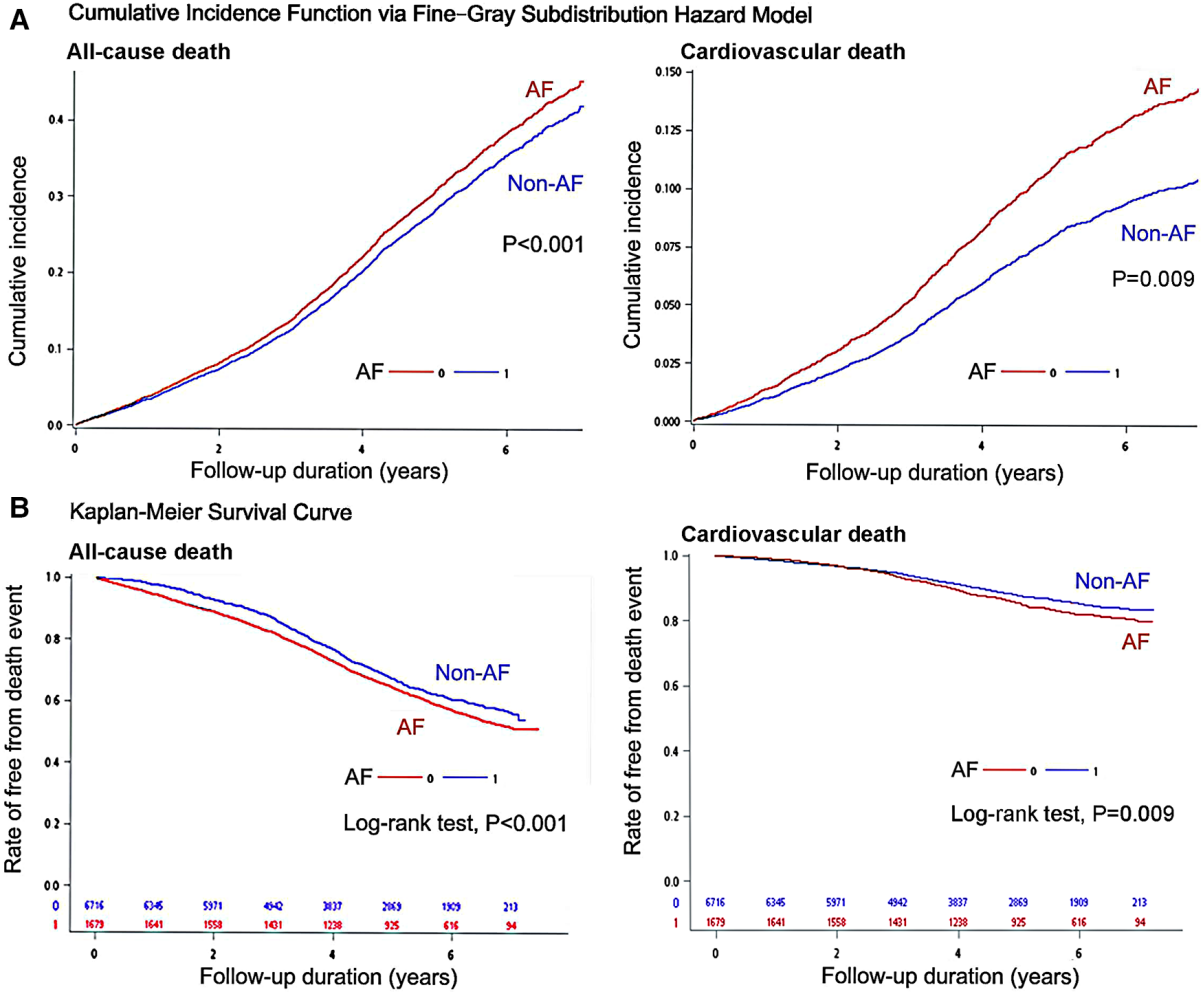
Survival analyses of all-cause death and cardiovascular death for evaluating the difference between non-atrial fibrillation (AF) controls and AF patients: (**A**) cumulative incidence function *via* fine-gray subdistribution hazard model, and (**B**) Kaplan–Meier event-free survival curves with log-rank test.

Survival analyses of all-cause death and CV death for evaluating the difference between non-AF controls and AF patients were shown in Figure 2. The cumulative incidence function *via* Fine-Gray subdistribution hazard model for estimating the competing risk of CV death revealed that an AF history was associated significantly higher risk of incident CV death (Figure 2A; *P* = 0.009). In addition, the outcomes of all-cause death were evaluated using the Kaplan-Meier method, and patients with dementia and a history of AF had poor mortality outcome (Figure 2B) compared with non-AF controls (log-rank test: *P*-value < 0.001).

For patients with dementia, a history of AF was associated with a higher risk of all-cause death (adjusted HR: 1.208, 95%:1.142–1.277; *P* < 0.001; Table 2) and CV deaths (adjusted SHR: 1.185, 95% CI: 1.033–1.358; *P* < 0.001). The risks of all-cause death and CV deaths were significantly higher in dementia patients aged <65 years with a history of AF than in dementia patients aged ≥65 years with a history of AF (*P* for interaction between age and AF: *P* = 0.003 for all-cause death, and *P* = 0.038 for CV death; Table 2). In addition, mortality risks in the dementia cohort assessing based on the status of AF as time-varying covariate were reported in Supplementary Table S2. The estimates of the regression coefficients for the status of AF in Supplementary Table S2 are similar to those obtained from the conventional Cox model or the Fine-Gray model reported in Table 2.

### 3.3. Risk factors of all-cause deaths in dementia patients with atrial fibrillation

Patients with AF and dementia had higher risks of all-cause deaths due to higher age (HR: 1.17, 95% CI: 1.15–1.18, Table 3) and CHA_2_DS_2_-VASc score (HR: 1.73, 95% CI: 1.29–2.31), sex of male (HR: 2.08, 95% CI: 1.50–2.89), a history of congestive heart failure (HR: 1.10, 95% CI: 1.003–1.20), diabetes mellitus (HR: 1.34, 95% CI: 1.21–1.48), chronic kidney disease (HR: 1.70, 95% CI: 1.38–2.00), and prior stroke (HR: 1.33, 95% CI: 1.22–1.45). Regarding the effects of medication uses on reducing the risks of all-cause deaths, novel oral anticoagulants (NOACs, HR: 0.87, 95% CI: 0.80–0.92, Table 3) and anti-arrhythmic drugs (HR: 0.93, 95% CI: 0.85–0.99); especially for patients using the anti-arrhythmic drugs of Class Ic significantly reduced the risk of death in patients with AF and dementia (HR: 0.77, 95% CI: 0.66–0.90; *P* < 0.001). In contrast with dementia patients with AF, dementia patients without a history of AF had higher risk of all-cause deaths due to higher age (HR: 1.15, 95% CI: 1.14–1.16, Table 3) and CHA_2_DS_2_-VASc score (HR: 1.56, 95% CI: 1.34–1.81), sex of male (HR: 1.95, 95% CI: 1.65–2.30), a history of hypertension (HR: 1.94, 95% CI: 1.63–2.30) and diabetes mellitus (HR: 1.77, 95% CI: 1.47–2.14).

**Table 3:** Using Cox proportional hazard model to evaluate the risk of all-cause deaths in dementia patients with atrial fibrillation.

Variables	Non-AF history (*N* = 6,716)		With AF history (*N* = 1,679)	
	Multivariable model: Hazard ratio (95% CI)*	*P*-value	Multivariable model: Hazard ratio (95% CI)*	*P*-value
Age at baseline (years)	1.150 (1.143–1.157)	<0.001	1.165 (1.148–1.182)	<0.001
Sex: male	1.947 (1.648–2.301)	<0.001	2.079 (1.498–2.886)	<0.001
CHA_2_DS_2_-VASc	1.556 (1.340–1.808)	<0.001	1.728 (1.291–2.313)	<0.001
AF duration (years)	Na	Na	1.001 (0.983–1.020)	0.88
**Underlying diseases**				
Hypertension	1.938 (1.631–2.304)	<0.001	0.923 (0.787–1.013)	0.09
Congestive heart failure	0.771 (0.566–1.050)	0.10	1.095 (1.003–1.196)	0.038
Diabetes mellitus	1.770 (1.466–2.141)	<0.001	1.341 (1.212–1.483)	<0.001
Chronic kidney disease	1.204 (0.945–1.534)	0.13	1.697 (1.375–2.003)	<0.001
Chronic obstructive pulmonary disease	0.914 (0.774–1.080)	0.29	1.043 (0.798–1.363)	0.76
Prior stroke	0.985 (0.904–1.072)	0.72	1.332 (1.215–1.453)	<0.001
Coronary artery disease	0.398 (0.149–1.060)	0.07	0.973 (0.812–1.164)	0.53
Receiving AF ablation	Na	Na	0.705 (0.388–1.277)	0.23
**Medication uses**				
Anti-arrhythmic drugs	0.872 (0.720–1.057)	0.16	0.927 (0.853–0.990)	0.029
Beta-blocks	1.012 (0.927–1.104)	0.79	1.141 (0.969–1.343)	0.11
Novel oral anticoagulants	1.026 (0.861–1.233)	0.73	0.868 (0.798–0.923)	<0.001
Warfarin	14.80 (0.961–228.3)	0.053	1.011 (0.993–1.021)	0.88

AF, atrial fibrillation; CI, confidence interval; *N*, number; Na, not available; HR, hazard ratio.

* Multivariable adjustment in the full model, including: age, sex, CHA_2_DS_2_-VASc, AF duration (years), underlying diseases provided in this table, status of AF ablation, and medication uses of beta-blockers, antiarrhythmic drugs, novel oral anticoagulants, and warfarin.

### 3.4. Risk factors of cardiovascular deaths in dementia patients with atrial fibrillation

Patients with AF and dementia had higher risks of CV deaths due to higher age (SHR: 1.16, 95% CI: 1.14–1.19 and CHA_2_DS_2_-VASc score (HR: 1.78, 95% CI: 1.13–2.81, Table 4), male (HR: 1.94, 95% CI: 1.15–3.30), a history of congestive heart failure (SHR: 1.31, 95% CI: 1.15–1.51) and prior stroke (SHR: 1.47, 95% CI: 1.29–1.72). Regarding the effects of medication uses on reducing the risks of CV deaths, only the use of anti-arrhythmic drugs significantly reduced the risk of death in AF patients with dementia (SHR: 0.89, 95% CI: 0.65–0.998, Table 4), especially for patients using the anti-arrhythmic drugs of Class Ic (SHR: 0.65, 95% CI: 0.50–0.84; *P* < 0.001). In contrast with dementia patients with AF, dementia patients without a history of AF had higher risk of CV deaths due to higher age (HR: 1.12, 95% CI: 1.11–1.13, Table 4) and CHA_2_DS_2_-VASc score (HR: 1.37, 95% CI: 1.02–1.83), sex of male (HR: 1.44, 95% CI: 1.04–1.99), a history of hypertension (HR: 1.49, 95% CI: 1.07–2.07, Table 4) and diabetes mellitus (HR: 1.54, 95% CI: 1.07–2.22).

**Table 4:** Using fine-gray sub-distribution hazard model to evaluate the risk of cardiovascular deaths in dementia patients with atrial fibrillation.

Variables	Non-AF history (*N* = 6,716)		With AF history (*N* = 1,679)	
	Multivariable model: Hazard ratio (95% CI)*	*P*-value	Multivariable model: Hazard ratio (95% CI)*	*P*-value
Age at baseline (years)	1.120 (1.107–1.133)	<0.001	1.161 (1.137–1.191)	<0.001
Sex: male	1.439 (1.040–1.992)	0.028	1.943 (1.145–3.297)	0.014
CHA_2_DS_2_-VASc	1.366 (1.021–1.828)	0.036	1.784 (1.132–2.810)	0.013
AF duration (years)	Na	Na	1.020 (0.990–1.051)	0.18
Underlying diseases				
Hypertension	1.486 (1.068–2.066)	0.019	1.116 (0.857–1.398)	0.28
Congestive heart failure	0.624 (0.343–1.134)	0.12	1.313 (1.145–1.505)	<0.001
Diabetes mellitus	1.541 (1.071–2.217)	0.020	0.615 (0.316–1.198)	0.15
Chronic kidney disease	0.903 (0.529–1.542)	0.71	1.000 (0.515–1.943)	>0.99
Chronic obstructive pulmonary disease	0.690 (0.472–1.008)	0.06	1.047 (0.681–1.611)	0.83
Prior stroke	0.727 (0.400–1.322)	0.30	1.473 (1.288–1.719)	<0.001
Coronary artery disease	0.477 (0.064–3.546)	0.47	1.297 (0.981–2.111)	0.15
Receiving AF ablation	Na	Na	0.847 (0.445–1.613)	0.61
Medication uses				
Anti-arrhythmic drugs	1.019 (0.727–1.427)	0.91	0.893 (0.649–0.998)	0.043
Beta-blocks	1.112 (0.948–1.305)	0.19	1.114 (0.841–1.475)	0.45
Novel oral anticoagulants	1.213 (0.980–1.823)	0.13	1.272 (0.949–1.705)	0.11
Warfarin	1.461 (1.065–2.005)	0.019	1.370 (0.997–1.764)	0.08

AF, atrial fibrillation; CI, confidence interval; *N*, number; Na, not available; SHR, sub-distributional hazard ratio.

For estimating the SHR, competing risk of cardiovascular death was evaluated using Fine-Gray subdistribution hazard model: Non-death (no death event: 0) vs. cardiovascular deaths (main event: 1) vs. non-cardiovascular deaths (other death event: 2).*Multivariable adjustment in the full model, including: age, sex, CHA_2_DS_2_-VASc, AF duration (years), underlying diseases provided in this table, status of AF ablation, and medication uses of beta-blockers, antiarrhythmic drugs, novel oral anticoagulants, and warfarin.

## 4. Discussion

### 4.1. Main findings

The main findings of this study were as follows: first, AF patients had a higher prevalence of vascular dementia than non-AF controls; and second, AF is an essential risk factor for CV deaths in patients with dementia. A history of AF increased the risk of all-cause death by 1.21-fold in patients with dementia. Especially for those who aged <65 years, risk of all-cause death increased to 5.55 times compared to 1.19 times for those who aged ≥65 years. Third, patients with a history of dementia were more likely to die from all causes if they were older, had congestive heart failure, diabetes, chronic kidney disease or a prior stroke; Last, medication uses of anti-arrhythmic drugs and NOACs reduced the risks of all-cause deaths for dementia patients with AF; however, medication use of warfarin increased the risk of CV deaths for dementia patients without AF.

### 4.2. Atrial fibrillation as a contributor to the mortality

In this study, we showed that the AF group had a higher prevalence of vascular dementia than the non-AF controls in dementia patients. Several large-population studies have reported that AF is associated with multiple adverse outcomes, including heart failure, stroke, and mortality (22, 23). Previous studies have shown that AF and dementia are frequently associated (8, 9). Patients with dementia are at risk for many medical complications and death due to mobility impairments (24). In contrast, AF is associated with impaired mobility in elderly individuals (25, 26) and may accelerate the risk of cognitive decline and lead to dementia (9). A meta-analysis showed that patients with AF had a higher risk of incident dementia than those without AF (27). The mechanisms of cognitive dysfunction and poor CV outcomes associated with AF include cerebral hypoperfusion, cerebral small vessel disease (e.g., cerebral thromboembolism and microbleeds), and systemic inflammation (28).

In several studies, higher ages, male sex, and severity of disease predicted higher death risks in dementia patients (5, 29); however, few studies have investigated these factors in dementia patients with AF. Our study found that dementia patients had higher risks of all-cause deaths due to older age, CHA_2_DS_2_-VASc score, male sex, and a history of diabetes mellitus irrespective of a history of AF. When only considering dementia patients with AF, they had higher risks of all-cause deaths due to a history of congestive heart failure, chronic kidney disease, and prior stroke; and dementia patients with AF had higher risks of CV deaths due to a history of congestive heart failure and prior stroke. In contrast with dementia patients with AF, dementia patients without a history of AF had higher risks of all-cause deaths and CV deaths due to older age, CHA_2_DS_2_-VASc score, male sex, a history of hypertension, and diabetes mellitus.

Previous research found that individuals with dementia and coexisting conditions of hypertension and diabetes may be at higher risks of mortality (30). Hypertension and AF are both critical concerns for public health. These conditions pose significant risks for cardiovascular diseases and mortality. Of these, hypertension is the most crucial risk factor that can be modified the occurrence of dementia and AF. Hypertension is considered a more important risk factor for mortality in dementia patients without AF compared to those with AF due to the different ways in which hypertension and AF impact cardiovascular health and contribute to death (31, 32). Hypertension can increase the risk of mortality by damaging blood vessels and increasing the risk of cardiovascular events (33), such as AF, heart attack and stroke, which can lead to death. In addition, hypertension can also worsen the underlying dementia and contribute to functional decline and decreased quality of life.

On the other hand, AF is a strong independent risk factor for mortality in dementia patients. This is because AF increases the risk of stroke and other cardiovascular events, which can lead to death. AF is also associated with other comorbidities, such as heart failure, which can further increase the risk of mortality. Our study revealed that AF patients with prior stroke were at a significantly higher risk of all-cause and CV deaths, which may explain the important role of cerebral thromboembolism as the key cause of mortality in dementia patients. In fact, stroke prevention is of paramount importance in managing AF patients. For the management of stroke risks in patients with AF, both European and American guidelines recommend to use the CHA_2_DS_2_-VASc scoring systems to determine an optimal strategy of stroke prevention (34). Higher CHA_2_DS_2_-VASc scores in AF patients were reported to be associated with increased risks of stroke, dementia (16), and mortality (35). In this study, higher CHA_2_DS_2_-VASc scores had a great impact on significantly increased risk of death in dementia patients with or without a history of AF. Our study findings suggest that while both AF and hypertension are important risk factors for mortality in dementia patients, the impact of hypertension on mortality may be greater in the absence of AF. However, further research is needed to better understand the complex relationship among AF, hypertension, and mortality in dementia patients.

### 4.3. Risks of mortality in patients with young onset dementia with atrial fibrillation

As previously mentioned, several studies indicated that as the age of dementia patients increases, their mortality risk increases (5, 29); however, the hazard ratio of mortality was the highest in the young age group and remained significantly elevated even in the older age groups (36, 37). Among dementia patients with a history of AF, CV deaths and all-cause deaths were significantly higher, especially among those younger than 65 years old. Individuals with dementia under the age of 65 are defined as having young-onset dementia, which can be classified into a number of subtypes with varying neuropathology and phenotype, and often presents with atypical symptoms, and is misdiagnosed and inadequate treated (38).

The death risks were significantly higher in younger dementia patients with AF than older dementia patients with AF could be due to several reasons (3, 5, 39): First, a diagnosis of young onset dementia has a unique impact, differing from that experienced by older individuals. Besides, prior research indicated that uncommon genetic variations-related arrhythmia may increase the likelihood of mortality in patients suffering from early-onset AF, particularly in those diagnosed at a young age (40). For individuals who are diagnosed with both young-onset dementia and AF, the condition tends to be more severe and progress rapidly, contributing to a higher mortality rate (37). Second, individuals with young onset dementia often face discrimination in reverse due to a lack of specialized services catering to their specific needs. Patients with young onset dementia are with less opportunity to achieve lifelong rehabilitation. These underlying causes may increase mortality rates. Our findings highlight the importance of proper diagnosis and management in younger dementia patients with a history of AF, and suggest that younger dementia patients with AF may require more specialized and targeted treatment and care, as their condition may progress differently and more rapidly compared to older individuals.

### 4.4. Effects of anti-arrhythmic drugs and NOACs in dementia patients with atrial fibrillation

Stroke is the most common consequence of AF; oral anticoagulant drugs remain the first-line medication for the prevention of ischemic stroke in patients with AF (9). In randomized controlled clinical trials, NOACs were associated with lower risk of stroke, intracranial hemorrhage, and death than warfarin in patients with AF (41, 42). In a nationwide population-based cohort study, AF patients receiving NOAC treatment had a lower risk of dementia than those who received warfarin (43). NOACs have shown better efficacy in preventing cerebral thromboembolism, and it is plausible that the use of NOACs could be associated with a lower mortality risk than the non-NOAC group in the present study.

Regarding pharmacological rhythm control in patients with AF, antiarrhythmic drugs of Class I or Class III were considered. Class Ic anti-arrhythmic drugs should be avoided in patients with ventricular dysfunction or a history of coronary artery disease. In contrast, Class III anti-arrhythmic drugs can be used safely in these patients, but with the risk of non-cardiac complications (44). Although some studies have reported that pharmacological rhythm control and rate control might not differ in the risk of CV deaths in patients with AF (45). Pharmacological rhythm control in AF patients aged ≥65 years with heart failure was associated with a lower risk of 1-year all-cause death (46). In addition, the effects of antiarrhythmic drugs on reducing the risk of death in dementia patients with a history of AF remain unclear. However, prior studies have shown that controlling cardiac rhythm (including medications for rhythm control and AF ablation) may be useful to reduce the risk of dementia in patients with AF (7, 47, 48). In the current study, we reported that the uses of anti-arrhythmic drugs may have potential benefits in dementia patients with a history of AF. However, only the use of anti-arrhythmic drug Class Ic was associated with reduced death risk in this study. In addition, we did not find that AF ablation was associated with reduced risk of death in dementia patients with AF, which may be due to the limited number of cases of AF ablation in this study.

### 4.5. Study limitation

The large number of population-based databases and the long-term follow-up were the strengths of our study. However, this study has some limitations. First, due to the nature of AF, the underlying characteristics (e.g., history of hypertension, heart failure, diabetes mellitus, and stroke) remained inconsistent after PS matching. Nevertheless, the distribution of CHA_2_DS_2−_VASc was similar between AF and non-AF groups. Besides, these potential confounders were further adjusted using multivariable Cox regression analysis. Second, there may have been diagnostic coding errors. Nevertheless, diagnostic data in the NHIRD were double-checked by a professional coding team at each hospital. Third, the NHIRD did not provide information regarding the type of AF persistence (paroxysmal or persistent AF), genetic data, lifestyle (e.g., diet, exercise, and smoking status), and environmental interactions, which may affect the outcomes. Lastly, the use of medications such as anti-arrhythmic drugs and anti-coagulants may affect the outcomes. However, changes in medication uses may occur over time due to the changing status of underlying diseases, and the effects of treatment should be further reassessed using randomized controlled trials with a proper sample size.

## 5. Conclusion

The prevalence of vascular dementia was higher in AF patients than in non-AF controls. AF is a risk factor of mortality in patients with dementia. The mortality rate of patients with dementia and AF is also affected by several risk factors, including older age, congestive heart failure, diabetes, chronic kidney disease and a prior stroke. In patients with dementia and AF, treatments with anti-arrhythmic drugs and NOACs are associated with a reduced risk of death from all causes.

## Data Availability

The data analyzed in this study is subject to the following licenses/restrictions: The datasets of the NHIRD for this study can be found in the Health and Welfare Data Science Center, Ministry of Health and Welfare; however, access to the NHIRD is restricted due to confidentiality clauses. Requests to access these datasets should be directed to https://dep.mohw.gov.tw/DOS/cp-5197-61332-113.html.
