# Supplementary material for: Atrial fibrillation as a contributor to the mortality in patients with dementia: A nationwide cohort study
Source: Front Cardiovasc Med. 2023 Apr 3;10:1082795. doi: 10.3389/fcvm.2023.1082795 (PMC10106772; doi:10.3389/fcvm.2023.1082795)
Supplement: Supplementary file 1 [file Table1.docx]

**Supplementary Material**

**Figure Captions**

**Figure S1:** Balance assessment: (A) Kernel density plot of the propensity score after matching, and (B) distribution of age at baseline, CHA_2_DS_2_-VASc score, and propensity score, between patients with atrial fibrillation (AF) and non-AF controls in the propensity score matched sample.


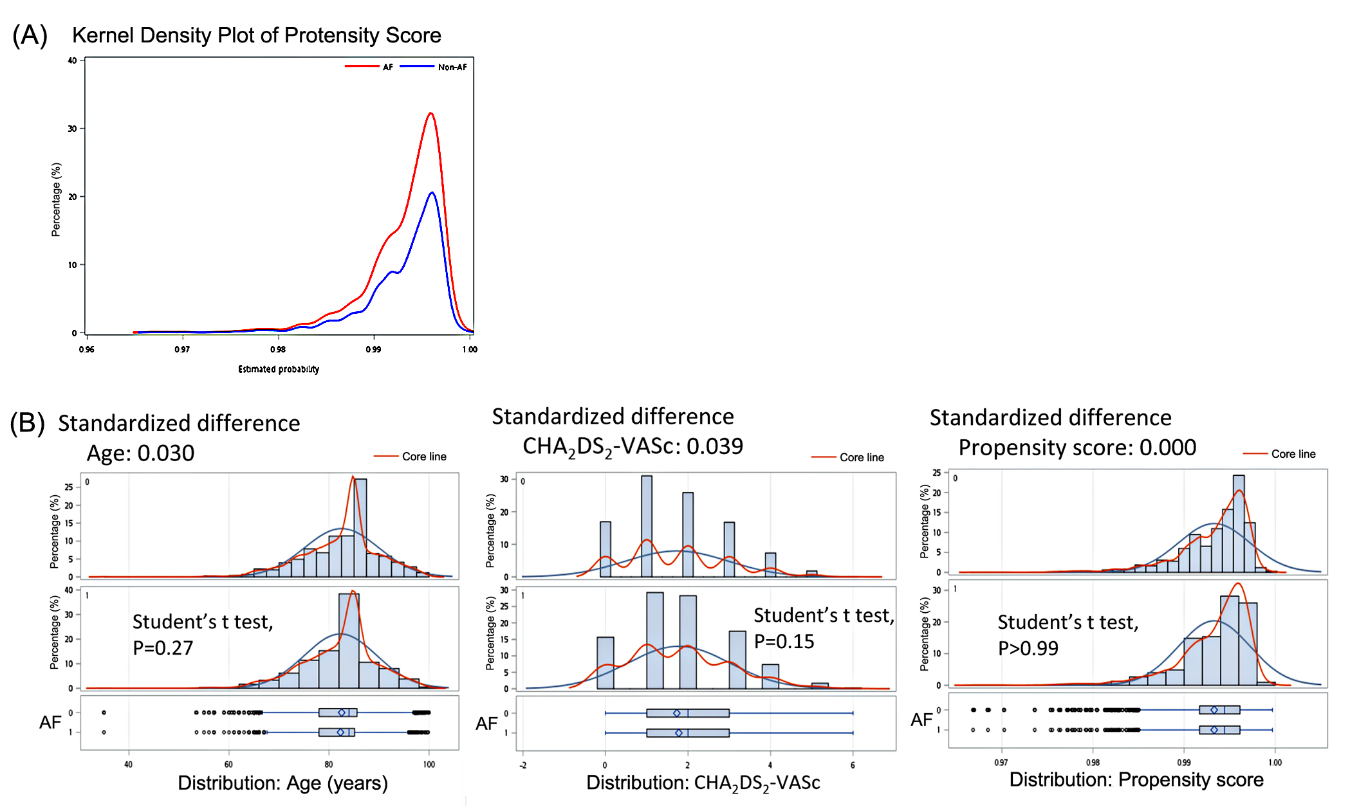


**Tables**

**Table S1: Baseline characteristics of patients with dementia (prior to propensity-score matching)**

| Baseline characteristics | Patients with dementia (N=335,189) | Non-AF history  (N=333,510) | With AF history  (N=1,679) | P-value |
| --- | --- | --- | --- | --- |
| Age at baseline (years) | 79.8±8.50 | 79.7±8.51 | 82.3±7.22 | <0.001 |
| CHA_2_DS_2_-VASc | 1.42±1.13 | 1.41±1.12 | 1.78±1.24 | <0.001 |
| Sex: men | 140,509 (41.9%) | 139,729 (41.9%) | 780 (46.5%) | <0.001 |
| Hypertension | 72,076 (21.5%) | 71,613 (21.5%) | 463 (27.6%) | <0.001 |
| Heart failure | 3,848 (1.15%) | 3,667 (1.10%) | 181 (10.8%) | <0.001 |
| Diabetes mellitus | 26,885 (8.02%) | 26,740 (8.02%) | 145 (8.64%) | 0.35 |
| Hyperlipidemia | 14,449 (4.31%) | 14,376 (4.31%) | 73 (4.35%) | 0.94 |
| Chronic kidney disease | 7,727 (2.31%) | 7,662 (2.30%) | 65 (3.87%) | <0.001 |
| Stroke | 50,472 (15.1%) | 49,965 (15.0%) | 507 (30.2%) | <0.001 |
| Coronary artery disease | 607 (0.18%) | 596 (0.18%) | 11 (0.66%) | <0.001 |
| Liver cirrhosis | 1,310 (0.39%) | 1,307 (0.39%) | 3 (0.18%) | 0.16 |
| Chronic obstructive pulmonary disease | 11,133 (3.32%) | 11,021 (3.30%) | 112 (6.67%) | <0.001 |
| Thyroid disease | 486 (0.14%) | 481 (0.14%) | 5 (0.30%) | 0.10 |
| Sleep apnea | 16,383 (4.89%) | 16,291 (4.88%) | 92 (5.48%0 | 0.26 |
| Receiving AF ablation | 158 (0.05%) | 0 (0.00%) | 158 (9.41%) | <0.001 |

AF: atrial fibrillation.

**Table S2: Mortality risks in the dementia cohort assessing based on the status of atrial fibrillation as time-varying covariate**

| Baseline characteristics | Without AF | AF as  time-varying covariate | P-value |
| --- | --- | --- | --- |
| Mortality outcomes | | | |
| Model 0: crude effect  (HR, 95% CI) | 1 (reference) | 1.094 (1.005-1.191) | 0.038^a^ |
| Model 1: adjusted effect*  (HR, 95% CI) | 1 (reference) | 1.144 (1.041-1.257) | 0.005^a^ |
| Age < 65 years*  (HR, 95% CI) | 1 (reference) | 5.549 (1.464-46.41) | <0.001^a^ |
| Age ≥ 65 years*  (HR, 95% CI) | 1 (reference) | 1.139 (1.037-1.252) | 0.007^a^ |
|  | P for interaction between age and AF: | | <0.001^b^ |
| Cardiovascular deaths (N, %) |  |  |  |
| Model 0: crude effect^Ɨ^  (SHR, 95% CI) | 1 (reference) | 1.339 (1.164-1.542) | <0.001^a^ |
| Model 1: adjusted effect*^Ɨ^  (SHR, 95% CI) | 1 (reference) | 1.242 (1.058-1.459) | 0.008^a^ |
| Age < 65 years*  (SHR, 95% CI) | 1 (reference) | 1.925 (1.042-68.12) | <0.001^a^ |
| Age ≥ 65 years*  (SHR, 95% CI) | 1 (reference) | 1.251 (1.066-1.468) | 0.006^a^ |
|  | P for interaction between age and AF: | | 0.022^b^ |

AF: atrial fibrillation; CI: confidence interval; HR: hazards ratio; N: number; SHR: sub-distributional hazard ratio.

Model 0: crude effect;

*Model 1 was adjusted for age, sex, CHA_2_DS_2_-VASc, chronic kidney disease, chronic obstructive pulmonary disease, beta-blockers, antiarrhythmic drugs, novel oral anticoagulants, and warfarin.

^Ɨ^For estimating the SHR, competing risk of cardiovascular death was evaluated using Fine-Gray subdistribution hazard model: Non-death (no death event: 0) v.s. cardiovascular deaths (main event: 1) v.s. non-cardiovascular deaths (other death event: 2).

Methods of statistical tests: ^a^ Cox proportional hazard regression, ^b^ interaction analyses.
